# *‘When will the doctor be around so that I come by?!’* Geo-socio effects on health care supply, access and utilisation: experiences from Kalangala Islands, Uganda

**DOI:** 10.1186/s12913-021-07204-7

**Published:** 2021-10-26

**Authors:** Japheth Nkiriyehe Kwiringira, James Mugisha, Mathias Akugizibwe, Paulino Ariho

**Affiliations:** Department of Sociology and Social Administration, Kyambogo University, Kampala, Uganda

**Keywords:** Geo-socio, Effects, Health, Care supply, Access, Utilization, Experiences Kalangala, Islands

## Abstract

**Background:**

The study set out to give an in-depth intersection of geo, eco-socio exposition of the factors relating to geography, healthcare supply and utilization in an island setting. This analysis is informed by what has emerged to be known as social epidemiology. We provide in-depth explanation of context to health care access, utilization and outcomes. We argue that health care delivery has multiple intersections that are experientially complex, multi-layered and multi-dimensional to the disadvantage of vulnerable population segments of society in the study area.

**Methods:**

We used a cross-sectional qualitative exploratory design. Qualitative methods facilitated an in-depth exploration and understanding of this island dispersed and peripheral setting. Data sources included a review of relevant literature and an ethnographic exploration of the lived experiences of community members while seeking and accessing health care. Data collection methods included in-depth interviews (IDI) from selected respondents, observation, focus group discussions (FGDs) and key informant interviews (KII).

**Results:**

We report based on the health care systems model which posits that, health care activities are diverse but interconnected in a complex way. The identified themes are; the role of geography, access (geographical and financial) to health services, demand and utilization, Supplies, staffing and logistical barriers and a permissive and transient society. When and how to travel for care was beyond a matter of having a health need/ being sick and need arising. A motivated workforce is as critical as health facilities themselves in determining healthcare outcomes.

**Conclusion:**

Geography doesn’t work and affect health outcomes in isolation. Measures that target only individuals will not be adequate to tackle health inequalities because aspects of the collective social group and physical environment may also need to be changed in order to reduce health variations.

## Background

One important dimension of health outcomes is where the person lives. Geographic disparities in health have been a concern [1, 2] indeed, *place* is considered a determinant of health more than vectors or micro-organisms [3]. Geographic disparities are associated with geographical accessibility. Geographical accessibility refers to the relative ease [4, 5] by which health services can be reached and utilized by a population when and where the services are needed [6, 7]. Thus, a shortage coupled with longer distances travelled by people to access health services may limit access to the services and cause possible geographic disparities in utilization of health services [8, 9]. Geographical access aims at analyzing the geographical dissemination of health care resources [10, 11]. In order to learn about the health of a population, the health of people within different geographical areas can be compared by equity, inclusion and access [12]. The explanation for geographical variations in health can be provided by socio-economic characteristics of an area. For instance, in Uganda, the north-eastern and island populations have less access to healthcare [13, 14], but the focus has been on technical factors in health care especially health infrastructure and supplies [15] and not geo-socio factors. Research on intersections of geo-socio health inequalities and exclusion factors have largely focused on HIV and AIDS among fish folk [16, 17], but not comprehensive health outcomes from an intersectionality framework. The geo-socio, service nexus in health care delivery for islanders has not been documented. In this study, we explored the intersectionality of geographical and social factors on healthcare. We provide an eco-social perspective to health provision and the production of ill health among islanders in Kalangala district. This study is a further exposition of what has emerged to be known as *social epidemiology* [18–20].

### Theoretical framework

Hill et al., [21] describe intersectionality as a way of understanding and analysing complex social conditions. In many cases, intersections are complex but skewed, multi-layered and multi-dimensional experiences [22, 23]. The study used intersectionality theory to understand the context and processes that shape both demand and supply of healthcare relating to lifestyle, care seeking and provision of health. Increasingly, intersectionality is seen as a good approach to the analysis of multifaceted processes that produce inequality and exclusion [24].

## Methods

### Study design

The study used a cross-sectional qualitative [16] exploratory design [25]. Qualitative methods facilitated an in-depth exploration and understanding of this island dispersed and peripheral setting. Data sources included a review of relevant literature and an ethnographic exploration of the lived experiences of community members while seeking and accessing health care. Data collection methods included in-depth interviews from selected respondents, observation, focus group discussions and Key informant interviews.

### Study area

Kalangala also known as the Ssese islands are 84 islands scattered in Lake Victoria with 65 habitable islands stretching near Kenya and Tanzania. These islands are served by only 15 health centres with only one health centre IV that serves as a regional health centre. The biggest Island is Bugala and covers an area of about 296Km2. Kalangala district is bordered by Mpigi district to the North, Mukono to the East, the united Republic of Tanzania to the South and Masaka and Rakai districts to the West [26, 27].

### Population and study sample

The study population were adult (males and females) and youth (in Uganda, youth are persons aged between 18 and 35 years) resident in Kalangala. No minors were interviewed. Data sources were district health officers; both technical and political including civil society and other service providers especially those in the health sector.

### Data collection

Graduate research assistants were trained and matched by sex with respondents. The development of data collection tools was informed by the recommendations of Johnston et al. [28] and Israel et al. [29] whereby as much as possible the mode of data collection was adapted for minimum disruptions on the study participants. Six (6) IDI were conducted in addition to 4 Key KI along with three (3) FGD for women, 3 for men and 3 for youth. Each FGD comprised of between 6 and 10 persons. Interviews did not last more than 90 min. We sought written consent before any interviews were conducted. Research assistants were graduates with social science research experience and familiar with qualitative research rigour and demands.

### Data management and analysis

Data cleaning and analysis were concurrent with data collection. For analysis, the local language versions of transcripts were first translated into English before importing the transcripts into NVivo version 11.0 for coding and analysis. Data was analyzed using content thematic approach, guided by themes contained in the data collection instruments. These themes were further refined following multiple readings of interview scripts to better understand the data. Typical quotations have been used in the presentation of findings. Identities of participants have been masked. We used concurrent triangulation during data analysis to ensure validity and reliability in the understanding of all intersecting issues.

### Ethical considerations

Ethical clearance was sought from the TASO REC (TASOREC/021/2020-UG-REC-009) which considered all ethical issues in the protocol. After review and approval, the proposal was again submitted to UNCST (SS506ES) for accreditation before data collection. Written informed consent was obtained from all study participants. All methods were performed in accordance with the relevant guidelines and regulations.

## Results

We report based on the health care systems model which posits that, health care activities are diverse, but interconnected in a complex way [30, 31]. The results are presented along the identified themes and subthemes as;

i) **The role of geography**

- Location as a barrier to service access
- Poor communication, transport and a transient population
- Seasons and healthcare

ii) **Access (geographical and financial) to health services, demand and utilization**

- Staff attributes and (ir)regularity
- Late opening and complicated access to health care facilities

iii) **Supplies, staffing and logistical barriers**

- Irregular medical supplies and stock outs
- Poor support supervision and poor health delivery
- Poor living and working conditions

iv) **A permissive and transient society**
v) Unusual generosity and unconventional sharing ‘*Ffe tulibalwadde*’ ‘we’re sick people -leave us alone’

- A risk economy and many cultures
- Unusual reasons to share HIV results

### The role of geography

#### Location as a barrier to service access

Kalangala consists of 84 islands, 56 of these have no health centers meaning that, many patients have to cross over from one island to another in search of healthcare. In addition, Kalangala district has no hospital. According to the Ministry of Health norms, to adequately cover its population of 54,100, the district should have a total of 16 health centers in addition to a hospital. Our findings indicate that health infrastructure in Kalangala is limited with the nearest hospitals in Entebbe (30-120 km) and Masaka (40-114 km). This geo-physical setting compromises the power of social capital, and social networks especially that communications, and transport means were unreliable. For far flung islands like Mazinga, and Bubeke, journeys to health centres were reported to take between 3 and 4 h by boat to access hospital level care. Poor health access was also linked to poverty especially those in hard-to-reach and hard to leave areas. Community outreaches were at times cancelled due to bad weather.

*Usually, there are extensive debates and considerations in view of the fear to get worse on the way; given the long journeys that are precarious including the fear to lose more than necessary along with the uncertainty of finding the health facility open including not being attended to … most locations in Kalangala did not have reliable telephone signals for timely communication …* District health official.

This complexity meant that, on one hand, the sick could not get timely attention, while on the other hand, health workers and supplies could not get to clients at facility level in time. On the whole, this precipitated poor health outcomes including high morbidity and mortality that were also poorly captured and documented due to underreporting given that people fell sick and died outside health facilities.

#### Poor communication and transport

The challenges of unpredictable transport sometimes made it impossible for the chronically and critically ill to keep clinical appointments including drug refills led to undesirable outcomes including dropping out of care. In some cases, such delays went up to a month of delay without treatment. This situation affected commuters that had two homes, one on the island and another off the island. Severe and chronic sicknesses were poorly handled in such locations including loss to care. Because of these transport and logistics related challenges, people usually showed up at the health centres when very sick. This increased both the morbidity that was caused by failure to get back for review as well as timely referral when non-responsive to treatment as well as mortality. Admittedly, there was very low health literacy as well as overall literacy and poor education indices on account of poor access, and delivery.

*‘Poor transport is compounded by the fact that, the safer modes of transport like ferries do not operate at night and yet many (health) emergencies present at night when time is of the essence* … ’ Private not for profit health service provider

Other risk multipliers on the island were related to small, slow water crafts that also leaked, overall poor marine safety due to lack of standards especially the lack of life jackets which increased the risk to passengers on case of an accident or need to evacuate the marine vessel. In the absence of public transport, travel means had been left to private providers that were motivated by profits making boat hire services prohibitively high for an average person. Because of the high costs of transport, community out reaches were sometimes cancelled. When community outreaches became interrupted, medications to the chronically could not be delivered. Transport was complicated by the substandard marine transport services and equipment mainly of canoes and poorly built boats. Safety concerns were paramount to service providers and yet, the health needs of the populace were dire. This dilemma especially the untimely delivery of supplies did not contribute to good health outcomes. Nkese Island near Kenya was specifically pointed out as having bad weather. The health outreaches to this island were the most unsuccessful. It was reported that there had been cases of losing clinical samples and supplies such as blood and testing kits after they fell in water due to turbulent waters.

*‘ … engines have fallen off boats. This is imminent danger... people have been lost for several hours including ending up in the wrong place sometimes resulting in running out of fuel. There are also cases of patients dying while transport is arranged especially that many usually fail to meet the emergency fare to referral facilities..!* Private Not for Profit Health care provider

As a coping mechanism, patients with chronic and terminal illnesses pooled money to travel as a group so as to cost share and reduce costs. In other cases, one person would be sent to collect supplies in the case of ARVs for the entire group. This in itself had associated risks and uncertainties including the possibility of mix-ups or a total loss of the package. However, the fear of disclosure and stigma had at times worked against such collective action in care seeking. In a bid to cope, whenever possible, especially for ANC and deliveries that were not emergencies, people planned and travelled early so as to avoid emergencies and last-minute travels that posed great uncertainty. Prior travel arrangements were beset by other associated costs of upkeep that not all could afford. Such and similar challenges inadvertently meant poor health delivery including loss to follow up with both chronic and terminal ailments getting worse. Kalangala being hard to reach and leave meant that, support supervision was also expensive, irregular and ineffective. This left many gaps in staff work hours and facility service times related to signing out for weekends earlier than expected (sometimes on Wednesday or Thursdays) and reporting back for the week on Monday or Tuesday. This was partly explainable from the lack of effective family services such as schools, housing and other life style amenities that made service providers migratory. For example, many civil servants had their families and investments on the mainland especially Masaka, Jinja, Rakai, Kampala, and Entebbe.

#### Seasons and health care

Treatment and care were affected by seasons and weather which made costs for transport expensive and sometimes impossible due to volatile waters.

*‘Even if you have all the money in the World, once the lake is turbulent, you cannot travel … here we only go for treatment when we have money because it is a season for fish and therefore, we have money, but also when the lake is calm. We have lost some people because the lake was not navigable … ’* Long stay resident/ islander.

For the chronically and critically ill, lives were at risk when bad weather impeded travel. It was reported that, both patients/ the general population as well as health care providers would at times be forced to cancel journeys to health centres to provide treatment and support on account of poor weather conditions. Geographical and locational factors such as these, significantly influenced health seeking and influenced health outcomes in an unplanned manner. Almost the same problems faced by the population, faced health workers as well. With a few exceptions, the further away the healthcare services, the lower their standards and use. While merely being close to a health facility doesn’t always guarantee better access, (especially due to cost and cultural factors), in Kalangala, the further away the facility was located, the more it was less likely to have qualified staff, no supplies and harder to be accessed. Over all, health care was worse for vulnerable groups and this was unnoticed and largely underreported.

### Access (geographical and financial) to health services, demand, and utilization

#### Staff attributes, (ir) regularity, availability and awareness of staff itinerary

Staff attributes such as being approachable and the knowledge about their qualifications meant a lot. The more staff were thought, perceived and known to be qualified (clinical officers, midwives and nurses) the more people would make it a point to seek services at such a facility. This was in addition to being reported to be available for duty by proxies and personal indicators; *‘Their car’*, the office/ house being open, being seen that day or week-end in town etc., the more people encouraged each other to seek care from these facilities (especially women and children). Staff availability was a key care seeking driver. There were indications that, the more staff were known to be qualified (clinical officers, midwives and nurses), the more people encouraged each other to seek care from these facilities Perceptions of health staff kindness and willingness to listen endeared patients not only to seek services, but also to wait and the willingness to postpone treatment and review ‘just to be seen by a particular health staff.’ ‘*When will the doctor be around so that I come by?!*’

#### Late opening and complicated access to health care facilities

Few and short opening hours were linked to lack of staff housing at health facilities, and long travel time that was made worse by bad weather with some staff absconding from duty. Although health worker deployment is estimated at 80% within the district, the working conditions had practically worked against these staffing norms.

*‘Most health facilities were closed on weekends, holidays, during bad weather, at night and for the most hours during the day because of late opening and early closing times … ’* Female FGD participant.

Without a risk allowance and poor facilitation, health workers in Kalangala faced a very expensive life which compromised their effectiveness. This reality made sickness an ordeal especially; when a partner or any person of help was away, when low on cash to hire a boat and more so, in an emergency or critical sickness or accident. This was so bearing in mind the likely long waiting hours at the health facility. Antenatal Care (ANC) was further complicated when a spouse would be required at the facility by health workers especially in dealing with Prevention of Mother to Child Transmission (PMTCT) cases and family planning among others. Such a requirement increased all costs of care and treatment. Spousal presence was also unattractive especially that men had many hustles out of which they made ends meet. Some men also perceived ANC as female spaces in which men were exempted. In response, care seeking was sometimes delayed or came at high cost on the household. These were at times pre-cursors to domestic violence, violence against women and sexual and gender-based violence.

### Supplies, staffing, and logistical barriers

#### Irregular medical supplies and stock outs

Irregular medical supplies were reported as common and contributing to non-adherence leading to poor treatment outcomes. Adherence and retention in chronic and terminal care were reported to be very difficult in a context of irregular supplies and stock outs. Irregular supplies affected both care seeking and health delivery in that, patients were reluctant to take long journeys without certainty of service especially that some trips were so expensive and required in excess of 100 l of fuel to travel in the case of private boat hire, such cost estimates were to the tune of about UGX 300,000/− (about US$ 85.00). Transport difficulties were most dire on the islands of Mazinga, Bubeke, and Jana. The results were poor health outcomes in form of late referrals, worsening conditions that would have been treated at less cost, permanent impairment and sometimes death.

#### Poor support supervision and poor health delivery

The geographical location of Kalangala islands also meant that support supervision was expensive, irregular and ineffective. This left many gaps in staff work hours and facility service times related to signing out and absence on weekends earlier than expected; (sometimes on Wednesday or Thursdays) and reporting back for duty on Monday or Tuesday of the next week. This was explainable given that a number of technical staff had their families and interests on the main land and therefore, had divided time between the work station -Kalangala and other concerns off the island. It was also reported that;

*‘For whoever got a breakthrough (financial or career -education related) they quickly migrated to the mainland where they perceived more and better opportunity and hence less investment on the islands.’* Local government official

This process had negatively affected the island service delivery and broader investment potential. Due to poor working conditions and weak support supervision, most diagnosis and treatment were provided by junior staff at nursing officer level and below, who treated patients based on *symptoms than diagnosis*. Because of this, asymptomatic cases were missed in treatment. This also led to delayed referrals and the associated complications.

#### Poor living and working conditions

The poor living conditions were partly because islanders regarded the fishing communities as temporary places to work rather than places to improve. This was especially due to the high-water table which made the delivery of good sanitation and proper environmental health costly in terms of water borne systems and associated costs. Respondents reported poor hygiene (that was also evident through observation) with the associated diarrheal diseases. Poor waste management was widespread coupled with pollution from dumpsites and burning of refuse in this otherwise pristine getaway to white sand beaches that were out of reach for the local residents. Majority of dwelling units were congested, small, multi-purpose and poorly lit; wood, cardboards, mud and wattle structures which compromised the essence of ‘housing’ as a basic need. This housing provision was not able to provide cover from extreme conditions for all age groups and population categories. This in itself meant that young children and the infirm would not get request conditions to recuperate in such kinds of shelter. Therefore, for the low-income earner, some discomfort and the lack of care and comfort were permissible. This was partly due to the unpredictable income streams that were weather dependent. The crackdown on illegal fishing hurt many livelihoods by cutting off incomes for many households and related service providers. This was especially that there were no alternative income measures or the provision of social safety-nets. The poor living conditions were further linked to the socio-demographics including household poverty and poor service delivery. Services such as safe water, sanitation, health and education facilities were often poor or entirely absent. Demands of the fishing industry, the strong occupational identities and incentives to remain in fishing negatively affected the educational outcomes of formal schooling.

### A permissive and transient society

#### Unusual generosity and unconventional sharing ‘Ffe tulibalwadde’ ‘we’re sick people -leave us alone’

It was reported in a male FGDs that women *belonged to the wider community*. This notion of women as *‘a common wealth’* was related to the uncertainty of life with men arguing that when they go fishing, they are neither sure when nor whether they will comeback. They therefore argued that, instead of letting their women starve while they are away, it was pragmatic to *‘let someone else take charge’* and look after their women. One quipped that, there was *‘no point in restricting another persons’* opportunity when you’re away …! There was a sense of *‘brotherhood’* as regards sexual partners on the islands.

‘The reality that fishermen did not move with their wives made them readily available husbands to females at the landing site. Whoever was present was the husband.’

*District official.*

*‘You cannot guarantee that I am going to the lake and I will comeback. None is sure!’*

IDI Fisher man.

While this was the view held by men, it was interesting that women were equally unbothered about the prospect of entertaining another man while the man of the house was away. Women in a FGD asserted that;

*‘ … you only welcome your partner when you see him! He also knows this! It is ok if he finds you not ready for him. He knows that everyone does this!’*

Men equally posited that; *‘.*

*Suppose I die? What will happen to her? Women cannot separate us; instead, women should unite us...’* Male Key informant

*‘A woman here has about five (5) husbands and this is normal. There is no worry about this … ’* KII -Local Government official

Such practices were reported among those in vibrant fish trade and therefore having regular and reliable, adequate income streams. It was argued that most fishermen were used to this life style of living in the moment. Life was simple, without a care.

*‘Every morning is just another day and the lake will provide fish and therefore money. There is no reason to worry and stress out.* Male boat owner.

*If there is a lake, fish, boats and men, your needs are sorted!*

IDI -long a female resident.

*‘If a woman stays in one place for about 3 months, she loses value … you need to meet new people. As such you must go to a new place so as to gain value.’*

Female Commercial Sex Worker.

*‘Fishermen here bet on the first person to sleep with a new woman in an area.’*

KII -Health worker.

*After everyman on an island has used you, you lose value. As such, you find a coping mechanism and have to move elsewhere to gain value since you are new after you have repackaged your identity.’*

Female Commercial Sex Worker.

In this way, prostitution is a livelihood in its own right. It was unique that, there was a form and type of ‘culture’ that allowed for being liberal with partners. This acculturation has been extended to the non-fishing community and residents on the island.

*‘Even us who are educated and delivering services in this place, we have many challenges at family level. The mainland is very far; our families are not here. As a result, we have developed a form of ‘divided affection’ between our families back home and the women here know this. For instance, over the weekend, University students from Nkumba storm this place dressed to kill. Campus students have made this a routine to come and make money from this place as we also spend more time with them. These ‘flowers’ are very popular and famous among both salary earners and successful fishermen. ‘Working here greatly changes one’s perspective of life. There is a totally different culture here.*

*It is very difficult to be faithful in this place.’* Male Civil servant.

Attractive females in the area are called ‘flowers’ that were to be ‘*visited, plucked and smelled*’. Men reported that, trust seemed to increase with females they knew less about and had no reason to ascribe sexual risk to them. Surprisingly, while fishermen did not care much about safe sexual practices, they equally asserted that, they know that they’re sick. This was because of what they knew about the causes of HIV. However, they also knew that, life is short and therefore to be enjoyed. Health workers reported that unlike other locations, *‘a negative HIV result was most unbelievable and always doubted … than a positive one … ’* Health workers indicated that;

*‘It took a lot of counselling and persuasion to let a person take a negative result as true. Those that were able, went on to carry out other tests to be sure about such an unlikely test result.’* Health service provider

#### A risk economy and many cultures

In Kalangala, risk had been normalized in all aspects of life including the norming of hyper risk episodes to include sexual relations. People were liberal in all aspects of life including being seen naked or having sex without privacy. On the whole, there was no secrecy about sex. It was reported that unprotected sex went for an entry price of Ugx 20,000, (about US$5.00) whereas protected sex was negotiable from about Ugx 5000 (about US$1.3). There were many teenage and adolescent mothers, many school dropouts and single parents. This was partly due to the attractive lifestyle associated with cash derived from fishing and commercial sex work as seen in those that dropout of school. Lack of progression in school was also linked to the inadequate adolescent friendly services on the islands largely because the environment is not attractive to skilled health workers. The islands were also a multi-cultural setting with many tribes and cultures with people from across the east African region.

*‘Here we have almost everyone, including people from Tanzania, Kenya, Rwanda, Burundi, Congo, Sudan, South Sudan, Somalia, and from all over Uganda that make socialization complex’* … Local council leader

The multiple ethnicities were amidst limited health services as well as other social service and primary healthcare deficits that partly associated with why HIV was first reported on these islands in Uganda.

#### Unusual reasons to share HIV test results

While HIV results and personal information are meant to be confidential, there were cases of volunteering to share test results among colleagues in an effort to clear doubts about one’s HIV status. This was the case especially when one wanted to win the favor of a prospective partner or dispel a rumor about one’s health status to serve as a *baseline* and basis for trust and acceptance among those they valued or courted. In other cases, people volunteered to share not their HIV test results, but the results of others as a form of *‘social responsibility.’* Such persons argued that *‘if you see a pit, you warn colleagues.’* While this was unethical, the perception was that since, test services were inaccessible (far and irregular) part of being a brother’ keeper was to ‘share’ such information among people one cares about. For those whose privacy was violated, this was a source of stigma. Because negative HIV test results were seldom, a negative result was peculiar and inadvertently increased ones sexual ‘value’. There were reports of some people seeking to obtain negative test results through bribery and falsification of results. This was one of the challenges related to the reliability of test results. The full extent of such practices was not established.

## Discussion

We argue that, while there’s a web of causation to poor health outcomes as a result of geo-social factors [32], there should be much more effort in finding the *spider* causing this web. Table 1 presents the mapping of these geo-social challenges as well as proposed recommendations.

**Table 1:** Challenges, exclusion, health outcomes and recommendations

Category	Source of Exclusion	Health Outcomes	Recommendations	Reviewed Literature
The uneducated especially Adolescents and youth	Poor health outcomes related to education being despised in favour of quick returns from fishing ‘having a partner with boats is a destination status …’ Negative peer influence and incorrect health information among the island community	Limited access to health information results in poor health seeking behaviour Early, teen and unplanned pregnancies Delay in testing for HIV and continued transmission of the virus	Appropriate sexual and reproductive health education Use positive role models to challenge negative norms Create micro-targets on each island to reach young people with clinical services Provide support services for key populations and link with CBOs Work with “gatekeepers/ and all stakeholders” to emphasize adolescent health, women welfare, education attainment and health as a means of personal and community development	Dingake, O. B. K. (2018) [33]. Engel, D. M. C., Paul, M., Chalasani, S., Gonsalves, L., Ross, D. A., Chandra-Mouli, V., & Ferguson, B. J. (2019) [34]. Ong, K. K. X., Ng, J. S., Om, C., Chhoun, P., Tuot, S., & Yi, S. (2020) [35]. Ali, M., Cordero, J. P., Khan, F., & Folz, R. (2019).
The uneducated especially Adolescents and youth	Poor health outcomes related to education being despised in favour of quick returns from fishing ‘having a partner with boats is a destination status …’ Negative peer influence and incorrect health information among the island community	Limited access to health information results in poor health seeking behaviour Early, teen and unplanned pregnancies Delay in testing for HIV and continued transmission of the virus	Appropriate sexual and reproductive health education Use positive role models to challenge negative norms Create micro-targets on each island to reach young people with clinical services Provide support services for key populations and link with CBOs Work with “gatekeepers/ and all stakeholders” to emphasize adolescent health, women welfare, education attainment and health as a means of personal and community development	Dingake, O. B. K. (2018) [33]. Engel, D. M. C., Paul, M., Chalasani, S., Gonsalves, L., Ross, D. A., Chandra-Mouli, V., & Ferguson, B. J. (2019) [34]. Ong, K. K. X., Ng, J. S., Om, C., Chhoun, P., Tuot, S., & Yi, S. (2020) [35]. Ali, M., Cordero, J. P., Khan, F., & Folz, R. (2019).
Pregnant Single Adolescents and Women in far flung islands	Negative cultural attitudes towards teenage pregnancy, estrangement and limited resources	Limited access to health services out of for fear of discrimination Consequences of termination of pregnancy leading to death	Recruit and train peer educators to serve as change agents Use social media for dissemination of youth-friendly health services Engage with communities on why delivering babies in health facilities is safer for both mother and baby Explore and adopt the use of technology and telemedicine for example the use of medical drones in remote locations Implement express of supplies and delivery for key populations.	Senior, K. A., & Chenhall, R. D. (2012) [36]. Woog, V., & Kågesten, A. (2017) [37]. Kennedy, E. C., Bulu, S., Harris, J., Humphreys, D., Malverus, J., & Gray, N. J. (2013) [38].
	Requirement of husbands/ spouses having to accompany pregnant woman to health facility for ANC Dependence on males/ spouses for permission and resources to attend clinic	Pregnant women without a willing partner are discriminated against and thus do not regularly attend ANC Pregnant women resort to bringing any man available which works against the intended benefits of male involvement Proxy husbands can result in GBV	Conduct Sexual and Reproductive Health Behavioural Change Communication (SRHBCC) targeting men to promote responsibility Empower women to be independent and able to access health care services. Invest in research that would cut costs of travel and delivery in terms of time and consumables. Upgrade the existing health facilities to be able to host and work with the new -proposed technology	Kakaire, O., Kaye, D. K., & Osinde, M. O. (2011) [39]. Kalisa, R., & Malande, O. O. (2016) [40]. Kaye, D. K., Kakaire, O., Nakimuli, A., Osinde, M. O., Mbalinda, S. N., & Kakande, N. (2014) [41]. Nkuoh, G. N., Meyer, D. J., Tih, P. M., & Nkfusai, J. (2010) [42]. Sialubanje, C., Massar, K., Hamer, D. H., & Ruiter, R. A. (2015) [43].
Sero- Positive persons	Internalized stigma Poverty and the lack of resources for transportation and health care services	Delay in seeking ARV treatment Take ARVS in hiding Do not have resources to get refills for ARVs	Explore alternative sites for distributions of ARVs and more practical modes of delivery. Increase income earning and focus on Poverty alleviation interventions through skilling, value chains and community infrastructure	Winchester, M. S., McGrath, J. W., Kaawa-Mafigiri, D., Namutiibwa, F., Ssendegye, G., Nalwoga, A., & Rwabukwali, C. (2017) [44].
Male Youth	Negative masculinity norms: fear of betraying culture especially letting women take control in decision making Preference for traditional healers on account of confidentiality, accessibility and negotiated payment terms. Leave school to fish	Men seek health services when they are in gross pain and the medical condition has progressed Alcohol and drug abuse Do not disclose full information Negative resilience and masculinities.	Mobile services and community outreaches Explore the viability of Health camps Conduct community level ‘test and treat’ outreach campaigns for the most at-risk key populations. Explore the use of male peers to promote appropriate male involvement and reach males	Easton, S. D., Saltzman, L. Y., & Willis, D. G. (2014) [45]. Lehman, P. (2007) [46]. Fleming, P. J., Lee, J. G., & Dworkin, S. L. (2014) [47]. Sileo, K. M., Fielding-Miller, R., Dworkin, S. L., & Fleming, P. J. (2019) [48].

From Table 1, the highlighted challenges resulted in poor service delivery, access, utilization and poor follow-up and lost care in case of chronic and life-threatening situations with frequent resort to self-medication [49], and polypharmacy [50]. Other undesired outcomes were giving up prescription medicine to a friend or relative including the *‘borrowing’* of medicines in order to avoid the inconvenience and costs of treatment. The reported *pharmaceutical altruism* was occasioned by running out of medicine by an acquaintance that was not able to afford accessibility and treatment costs, emergency situations and the convenience of sharing. We found a social context of medicine sharing relating to the same reasons why people shared [51] other resources.

When and how to travel for care was beyond a matter of having a health need/ being sick and need arising [52–55]. Rather, it was when the means and conditions permitted in view of weather, availability of means and money [55–57] as well as opening hours at the health facility and the odds of finding a health worker. Even when there were emergencies, it was not easy to get care. Weather and the season on the lake and fish catches meant having money or not; being broke or having liquidity [58, 59]. In this sense, emergency care was least effective since very sick people cannot hold out for about three hours on rough waters. Because of this, emergency and chronic diseases that would have otherwise been managed became fatalities and in some cases becoming sources of disability and complications [60, 61]. One common example was prolonged labour and cases of obstetric fistula. The situation on the islands was made worse by a poor transport system, characterized by irregular transport services and boats that were in poor conditions especially being substandard [56, 62].

While society tends to place equal responsibility on individuals for compositional health risks, it offers inequality [1, 63] in the means to deal with them. This was illustrated by the exotic tourist destinations on Ssese islands that are sought after places by the high-class and the elite. However, these locations are private and not typical of the average life style on the islands. The tourist resorts are insulated against the everyday limitations of the islanders through the use of speedboats, health checkups including air ambulance services among other composite features afforded by the wealthy.

### Intersectionality in perspective

Where a person lives, is as important as who they are in terms of health [64–66]. Geography, culture, context, health and place [12] especially income, education, relations, beliefs and infrastructure/ attitudes/ leisure have different meanings attached to life and in a given place [20, 67]. In Kalangala, *Kulya sente kivubi* meant living in the moment and *for the moment*. It was widely held that, the riskier episodes one experienced and survived; the more risk appetite one seemed to get and be aloof to safety. Such findings have been previously reported among fishing communities [58]. In all ways, there was no need and never a worry about tomorrow [58, 68]. While this was not new, it had a more dramatic effect in Kalangala. This mindset did not consider poor health or any other cause of future failed income. Individuals and households are molded and influenced by their local environment. Local circumstances are key in understanding people’ health [16, 24, 69]. Where you live, work, play largely explains the choices made and place explains a lot of this socio-spatial component since place exerts an influence on a range of health outcomes e.g., smoking and alcohol consumption among fishermen [58, 70]. At micro-level, individual/household and community factors also affected access and use of health services. This is the context of health care in terms of access and affordability [71].

Unreliable, unpredictable, unsafe and unaffordable transport [72–74] resulted in poor follow-up and lost care in case of chronic diseases and life-threatening situations with frequent resort to self-medication, and polypharmacy [75]. Other effects were the giving of prescription medicine to a friend or relative including the ‘*borrowing’* of medicines in order to avoid the inconvenience and costs of treatment, so long as they had the same symptoms and symptom matching, past illness experience [76] or believed that the one sharing had an idea about the disease at hand or knowledge about the medicine [77]. This resulted in high risks of drug resistance and poor recovery by patients who faced a number of financial and geographical challenges.

While geographical access in the country has improved [78], Kalangala district had few health facilities. Even with income earning opportunities in the fishing economy, the living conditions were generally poor. This was common for most landing sites in Uganda. Overall, this negatively affected the lives and health outcomes of fisher folk and islanders especially due to unsanitary living and crowding. The various service delivery challenges on Kalangala islands need service modes that are not contingent on time of day, weather and season or being well-off or not. Overall, other aspects of life; home, work and school (it was quite easy to drop out of school) were also dependent on weather, and seasons. On account of geography, far off islanders that needed better care on the main land, bicycles, motor cycles and vehicles did not have a significant role in care as is the case for other parts of the country. And yet, private boat ownership was not possible for almost the entire population. This made medical emergencies and referrals very unique and much more complicated than in other parts of the country. This makes Kalangala unique in all aspects of health care and calls into discussion the need for affordable public transport as part of the health delivery model in Kalangala.

Physical access and personal welfare for health care staff including housing are important factors in health care provision, access and utilization by the population on a regular basis. Private facilities closed the gap for those that can afford (the well-off), but also where they existed; their access was by affordability and not spatial proximity [79, 80]. Private facilities had an income barrier, at utilization than at access, while most public facilities had long distance as barrier [81]. Therefore, private health facilities are crucial in improving health access if the affordability issue is addressed. This is a recourse to welfare improvements and poverty reduction [82] in health care delivery.

Knowing someone (irrespective of rank and title) at the public health facility (a guard or even a cleaner) was perceived as useful since they relied important information such as availability of drug stocks, presence of staff and generally when to seek better services. This was a form of social capital and a very big asset. The longtime lost in travelling especially for pregnant and lactating mothers was including the various costs associated to access and receiving treatment, acquiring medicines and returning home. Such costs were often a disincentive to care-seeking among poor people [83] even when medicine were available and free. While women have traditionally been the ones keener to seek health care than men [84], the picture in Kalangala was different. The transport challenges had a gender dimension to access and affordability. There have been indications that out-of-pocket payments are inequitable and exploitative given that they aggravate poverty with likely poor treatment outcomes [85, 86]. In the case of chronic sickness, out of pocket costs more often led to disposal of assets such as fishing nets and boats. Where this was not possible, the other option was the acquisition of debt. Similar findings have been reported elsewhere [87, 88].

We posit that, individuals do not exist in isolation but, rather in places within the environments and spaces [89, 90] that are affected by seasons [91] as being favorable or not. This recognizes that, people exist in complex setups that create and shape health [92–94]. Individuals are not separate from the geo-social context with health outcomes as part of a broader system including how well responses to health needs [89] and demands are met. There is an urgent need to explore the interactions between all variables in order to understand the complexities of multiple causality in explaining health outcomes. We are convinced that, some places explain health inequalities more than others.

### Study limitations

This was a qualitative study that did not collect data from all islands in Kalangala district and yet, each island has its own unique features, ranging from income, culture and service infrastructure. Even then, this study offers a qualitative exposition of the geography -culture nexus of healthcare access, delivery and outcomes. These findings offer a thick description of the goings on in Kalangala that can be a basis for further inquiry. There is need to have a fully blown study that represents all islands in the district so as to inform actionable interventions for each island location based on quantitative evidence.

## Conclusion

Income and cost of care are crucial when a choice of care is being made. Physical access and personal welfare (income and education) are as well important factors in health care access and utilization. A motivated workforce is as critical as health facilities themselves in determining health care outcomes. It is useful in the long-run to treat the root causes, instead of focusing on symptoms. This is the call to see places as more than the sum of the current human populations living and dying within them. In a great detail, places form people as much as places are formed from people’s actions. Geography doesn’t work and affect health outcomes in isolation. Social status and livelihoods mediate the role of geography on health outcomes. Health variations, delivery, demand and access are a commentary on general welfare in a population. Measures that target only individuals will probably not be adequate to tackle health inequalities because aspects of the collective social group and physical environment need to be changed in order to reduce health variations.

## Data Availability

All the data sets that were used for this study are available and in case of need, they can be accessed from the corresponding author (Japheth Kwiringira –PhD) upon request.

## Abbreviations

ARVs: Anti-Retroviral
GBV: Gender Based Violence
ANC: Antenatal Care
SBCC: Sexual and Behavior Change Communication
RH/FP: Reproductive Health and Family Planning
CBOs: Community Based Organizations
PNFP: Private Not For Profit
CSW: Commercial Sex Worker
IDI: In-depth Interview
KII: Key Informant Interview
FGD: Focus Group Discussion
PMTCT: Prevention of Mother To Child Transmission
PHC: Primary Health Care
MCH: Maternal and Child Health
